# *Bacillus subtilis* Contributes to Amylase Production in the Honey Sac of *Apis mellifera*

**DOI:** 10.3390/insects16020221

**Published:** 2025-02-18

**Authors:** Miao Wang, Wenzheng Zhao, Danyin Zhou, Jian Huang

**Affiliations:** 1 College of Food Science and Technology, Yunnan Agricultural University, Kunming 650201, China; ynbee@163.com; 2 Faculty of Animal Science and Technology, Yunnan Agricultural University, Kunming 650201, China; 2014009@ynau.edu.cn (W.Z.); zdy@ynau.edu.cn (D.Z.)

**Keywords:** amylase values, honey sac, *Bacillus subtilis*, *Apis mellifera*, rape flowers, feeding experiments

## Abstract

Background: Amylase activity is a critical biomarker for assessing the freshness of honey. Historically, bees have been considered the sole source of honey amylase. However, recent studies suggest that *Bacillus subtilis* may also contribute to amylase production in the honey sac of *Apis mellifera*. Methods: In this study, amylase levels were measured in samples of nectar, honey sac fluid, and honey. The identification of *B. subtilis* in nectar, honey sac, and honey was evaluated. An in vitro bacterial culture system and a feeding experiment were developed to simulate honey sac conditions. Results: Our results showed that *B. subtilis* was detected in all sample groups, with the highest concentration in honey sac samples. Amylase levels in honey sac and honey samples were significantly higher than those in nectar. In the simulation experiment, amylase activity was only observed in cultures containing both *B. subtilis* and sucrose/nectar; no activity was detected in cultures containing only H_2_O or no *B. subtilis*. In the feeding experiment, bees fed sucrose or nectar showed higher amylase activity in their honey sacs than those fed water. Conclusions: Our data show that *B. subtilis* can produce amylase and offer potential for more standardized quality assessment of honey.

## Simple Summary

Amylase values in honey serve as a definitive biomarker for assessing the freshness of honey, with its concentration being a critical parameter for determining compliance with product standards. *Bacillus subtilis* contributes to amylase levels in honey sac fluid and honey, in addition to *Apis mellifera* secretion. Amylase activity in honey sac fluid exceeds nectar values, with the presence of *B. subtilis* correlating with higher amylase levels. In vitro experiments confirm *B. subtilis* amylase production in the presence of sucrose or honey sac fluid. Bacterial counts peak at 10^5^ CFU/mL in the honey sac, indicating a significant role in amylase synthesis. This study establishes a novel methodology for the analysis of bacterial amylase in honey, which may improve honey quality assessment. This discovery has important implications for the standardization and quality assessment of honey, suggesting that future regulations and freshness indicators should account for the role of microflora in addition to traditional measures.

## 1. Introduction

Amylase values in honey serve as a definitive biomarker for assessing the freshness of honey, with its concentration being a critical parameter for determining compliance with product standards [1,2]. α-Amylase in honey is one of the oldest studied honeybee enzymes. This enzyme is thought to be found in honey after being secreted from the secretory glands of honeybees. It has been isolated, purified, and studied for its various properties, including pH, temperature, and kinetics parameters [3]. Amylase values in honey are standardized as diastase activity, which is reported as diastase number and used as an indicator of honey freshness as the CODEX Standard [4,5]. The amylase content of honey is subject to degradation over time, particularly during prolonged storage [6,7]. In addition, the activity of honey amylase is modulated by a number of factors, including thermal treatment conditions, pH, the presence of metal ions, and the botanical origin of the nectar [8]. Collectively, amylase activity is an important predictor of honey quality and safety [9].

It is well known that during the honey production process, honeybees collect nectar, which enters the honey sac, forming a mixed solution of nectar, bacteria, and the bees’ own secretions. The nectar is only temporarily stored in the honey stomach (honey sac), and when the bee returns to the hive, it regurgitates the temporarily stored mixture back into the hive for further honey production [10].

The insect gut microbiome is a complex ecosystem with a diverse array of bacterial species that maintains a stable and dynamically balanced state after colonization. This microbiome is essential to the biology of the host insect and exerts a significant influence on its physiological processes and environmental adaptability; for example, the gut microbiota is known to promote pollen digestion in the worker honeybee, *A. mellifera*, thereby increasing longevity and body weight gain [11,12,13]. The autochthonous gut microbiota actively modulates host physiology, including the immune response, and confers resistance to pathogenic threats [14,15]. These bacteria, especially probiotics, are characterized by their biosynthetic capacity to produce essential nutrients that the host cannot synthesize independently. This symbiotic interaction is fundamental to the metabolic health and well-being of the host [16,17]. The honeybee gut microbiota is not only involved in the nutritional metabolism of bees, but may also influence their immune system and resistance to disease [18,19]. Previous studies have identified spore-forming bacteria such as *B. subtilis* and *B. amyloliquefaciens* in the honey sac, as well as other types of bacteria [20]. *B. subtilis*, which is abundant in the environment, is capable of producing amylases, lipases, and proteases that can degrade various chemical components, including lipids, proteins, and starch [21]. Therefore, in combination with the honey production process, we assumed that the bacteria in bees would also have some influence on the honey production process and further used them as a basis for bee product evaluation.

The role of bacterial amylase in honey has been largely overlooked, as it was previously thought that all amylase in honey came from bee secretions [22]. Our previous comparative analysis of amylase and bacteria in rape nectar and honey in the bee foregut showed that the amount of amylase in the bee foregut was significantly higher than that in the nectar, and this change was related to the increase in bacterial concentration. The addition of bacteria to the nectar confirmed that the increase in amylase was caused by bacteria, suggesting that bacteria in the bee foregut help to process the nectar into honey [23]. During nectar collection, the honey sac becomes filled with a liquid medium containing both bee-derived and bacterial-derived amylase, which is later regurgitated back into the hive for honey maturation [24,25]. Therefore, we hypothesize that the amylase levels may differ among nectar, honey sac, and honey due to the differences of *B. subtilis* in these three locations.

In this study, amylase levels were analyzed in the samples collected from nectar, honey sac, and honey. Additionally, the molecular and biochemical identifications of representative dominant bacteria in these samples was conducted. The results of this study aim to elucidate whether *B. subtilis* contributes to amylase activity in these three different locations. To determine if *B. subtilis* can produce amylase, an in vitro bacterial culture system was developed to simulate conditions in the honey sac. The same amount of *B. subtilis* as found in the honey sac was cultured in the presence of supplements such as H_2_O, sucrose, and nectar. Additionally, a feeding experiment was conducted to analyze amylase levels produced by *B. subtilis* in the honey sac of bees fed with either H_2_O, sucrose, or nectar.

The present study provides important insights for the standardization and quality assessment of honey. It is suggested that future regulatory frameworks and freshness indicators should include the influence of microflora in conjunction with conventional parameters. Such an approach is expected to facilitate the re-evaluation of the measurement criteria for honey freshness, thus having a profoundly beneficial impact on the healthy development of the industry from a scientific point of view.

## 2. Materials and Methods

### 2.1. Sample Collection

In the initial phase, we studied nectar source plants near an apiary, and there were no other flowering plants in the same period. In addition, *A. mellifera* was chosen as the experimental object because *A. mellifera* does not collect fewer nectar sources when the nectar source is sufficient but mainly uses a single nectar source. Rape flowers were collected near a bee apiary to better control the influence of the environment. The collection period spanned from the 12th to 23rd of February, 2021 to 2023. Nectar was gathered from rape flowers in a western honey bee apiary located in Luoping, Yunnan Province (24°57′ N, 104°30′ E, Altitude: 1666 m).

#### 2.1.1. Nectar Collection

Rape flower nectar was collected using a sterile capillary tube when the rape fields adjacent to the beehives were in full bloom. Approximately 50 mL of nectar was gathered from the same location. The collection was conducted under aseptic conditions using sterile micro-syringes.

#### 2.1.2. Collection of Honey Sacs

A total of ninety honey sacs were collected from three colonies, with thirty honey sacs from each colony. Specifically, for each colony, ten of the collected honey sacs were placed in a centrifuge tube containing five milliliters of sterile water, three tubes for each colony, making a total of nine tubes for three colonies.

#### 2.1.3. Honey Sac Fluid Collection

The honey sac fluid was extracted under sterile conditions from bees with nectar-filled sacs, using sterile micro-syringes for collection. Honey sac fluid was collected annually from three fixed colonies within the same apiary.

#### 2.1.4. Fresh Honey Collection

An empty honeycomb was inserted into each of the three *A. mellifera* colonies, and 5 mL of honey was collected from each colony using a sterile tube after 4 to 6 h. In total, 15 mL of fresh honey was collected from the three colonies that year. The colonies that produced fresh honey annually are the same ones that collect honey sacs.

#### 2.1.5. Sample Storage

All samples were immediately stored at 5 °C for subsequent experiments.

### 2.2. Bacterial Culture and Identification Assessment

#### 2.2.1. Methods for Culturing Bacteria

Using the conventional dilution culture method, 5 mL of nectar, honey sac, and honey samples were each added to 45 mL of sterile water and diluted according to a 1:10 gradient; then, the bacteria were isolated and identified. The experiment was repeated three times after diluting each sample with H_2_O; H_2_O was used as a blank control. Tryptone soy broth (TSB) agar was used as the culture medium in this study. The isolates were cultivated aerobically in TSB medium at 37 °C for 2 to 3 days. Colonies with distinct morphological characteristics were carefully selected, with 30 to 300 colonies chosen per plate. After initial isolation, these colonies were sub-cultured to obtain axenic cultures. This methodological approach facilitated the isolation and characterization of individual microbial strains, thereby enhancing the reliability of subsequent experimental analyses.

#### 2.2.2. Identification Assessment for *B. subtilis*

##### 16S rRNA Identification Methods for *Bacillus* Species

Bacterial thalli weighing between 0.1 and 0.3 g were harvested by centrifugation. Pure DNA was extracted using the Tianamp Bacteria DNA extraction kit (Tiangen, Biotech Co., Beijing, China).

Molecular identification of the isolates was performed by PCR amplification of the corresponding 16s rRNA genes followed by their sequencing. Briefly, the genomic DNA of the pure isolates was extracted using genomic DNA extraction kits (Tiangen, Biotech Co., Beijing, China), and PCR was performed using 27F (5′-AGAGTTTGATCCTGGCTC-3′) and 1387R (5′-GGGCGGTGTGTACAAGGC-3′) primers as forward and reverse universal 16S rDNA primers, respectively. PCR amplification of the 16S rRNA gene from each bacterial sample was performed using a thermal cycler (MJ Research, T100TM Thermal Cycler; Bio-Rad Co., Hercules, CA, USA). Each reaction mixture (final volume, 50 µL) contained 4 µL template DNA, 0.2 µL each primer, 25 µL 2× TransTaqTM II HiFi PCR SuperMix II (Transgen Co., Beijing, China), and 20.6 µL dH_2_O [26,27].

The purified PCR products obtained from the bacterial isolates were sequenced at Sangon Biotech Co. (Shanghai, China) using the 27F and 1387R primers. To identify the closest known relatives of the partial 16S rRNA gene sequences, the sequences were queried against GenBank (National Centre for Biotechnology Information, Rockville Pike, Bethesda, MD, USA) using the Basic Local Alignment Search Tool (BLAST; http://www.ncbi.nlm.nih.gov/, accessed on 12 July 2023). The National Center for Biotechnology Information (NCBI) database (accessed on 12 July 2023) and the BLAST search tool were used to identify the genus and species of the bacteria [28].

##### Determination of Biochemical Identification for *Bacillus* Species

Bacterial cultivation and processing: Bacterial strains belonging to the genus *Bacillus*, as determined by 16S rDNA sequencing, were grown in tryptic soy broth (TSB) medium at 37 °C for 2 to 3 days. Bacterial cells were harvested by centrifugation to achieve a McFarland standard 2 turbidity.

Biochemical identification of *Bacillus* sp.: The API 50 CH kit (bioMérieux, catalogue number: 50300F) was used for the biochemical characterization of *Bacillus* species following manufacture’s instruction.

Preparation of the incubation tray: A volume of 10 mL of sterile distilled water was added to the bottom of the incubation tray. The test strips were then placed in the tray. Using a sterile pipette, bacterial suspensions were inoculated into each of the 50 wells on the API 50 CHB/E test strips. Incubation was performed at a constant temperature of 37 °C in an incubator.

After 24 and 48 h of incubation, the test strips were read to interpret the biochemical reactions. Each test was scored and categorized as positive (+) and negative (−). The results were meticulously recorded on the test record sheets to create a biochemical reaction profile. These profiles were then entered into an automated system for microbial identification and antimicrobial susceptibility analysis. Definitive identification results were then obtained using *Bacillus*-specific identification software.

### 2.3. Analyses of Amylase Activity

Amylase (AMS) values were determined using an SFE90183 kit (Shanghai ShiFeng Biological Technology Co., Ltd., Shanghai, China). The substrate buffer (0.5 mL) was preheated in a water bath at 37 °C for 5 min. Then, 0.1 mL of the sample to be tested was added and the mixture was incubated in a water bath at 37 °C for 7.5 min. Subsequently, 0.5 mL of iodide reagent was added, followed by the addition of 3.0 mL of double-distilled water, whereas the blank control received an additional 0.1 mL of double-distilled water. The absorbance was measured at 600 nm using a spectrophotometer with a 1 cm path length to determine the optical density (OD) value. The experiment was repeated 5 times with H_2_O as a blank control for each nectar, honey sac fluid, and honey sample.

The calculation formula, which typically involves comparing the OD value of the sample with that of the blank control to calculate the activity of the enzyme in international units (IU) or similar units, is as follows:

$$AMS\left(U/dL\right)=\frac{Blank\;OD-Determination\;OD}{Blank\;OD}\times \frac{0.4\times 0.5\;}{10}\times \frac{30\;min}{7.5\;min}\times \frac{100\;}{0.1}\times Dilution\;ratio$$

### 2.4. In Vitro Simulation Experiment

The simulation experiment aimed to mimic the conditions in the honey sac and investigate whether *B. subtilis* can produce amylase. Bacteria were cultivated in tryptic soy broth (TSB) medium at 37 °C for 48 h. Spread plating was performed for enumeration, and the colony-forming units per gram (CFU/g) were calculated. *B. subtilis* cells were spun down by centrifugation, and 1 g of the pellet was subjected to gradient dilution. The same amount of *B. subtilis* as found in the honey sac was used. A variety of supplements were tested including H_2_O, 10% sucrose, 20% sucrose, 30% sucrose, and nectar with a sugar content of 12° Brix. In the control groups, no *B. subtilis* was added to each supplement. In the experimental group, *B. subtilis* bacterial cells were mixed with either H_2_O or 10% sucrose, or 20% sucrose, or 30% sucrose or nectar separately. Amylase activities were analyzed by using an SFE90183 kit as described previously.

### 2.5. Feeding Experiment

In this experiment, H_2_O, 10–30% sucrose solution, and nectar with a sugar content of 12° Brix were placed in the feeding tanks of 5 different colonies in the same apiary, and twigs were placed in the experimental solution to prevent the bees from drowning. After 1.5 h, the bees were removed from the feeding tanks, and the amylase levels were determined by absorbing the honey sac fluid [29,30]. The group of bees fed only with H_2_O served as the control group, and the amylase activity from this group was used as a baseline amylase level for the bee honey sac. Honey sac fluids were collected from bees and analyzed for amylase activities using an SFE90183 kit as described previously.

The amylase activity produced by *B. subtilis* in the honey sac was calculated by using the following formula: Amylase activity produced by *B. subtilis* in the honey sac = Amylase activity in the experimental group- Amylase activity in the control group.

**Figure 1:**
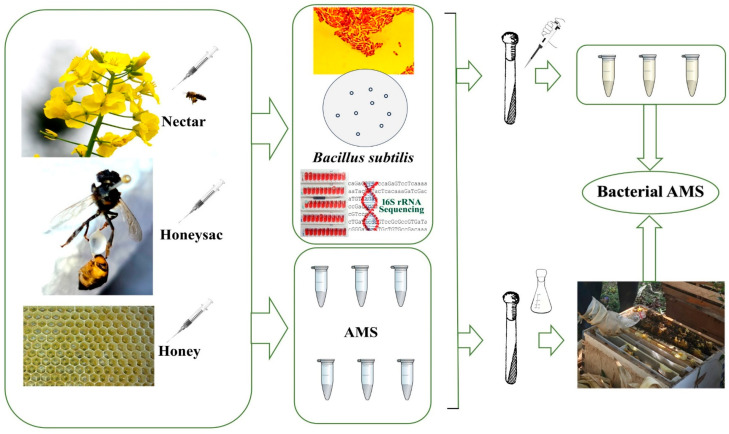
Schematic of sampling and experimental procedures.

As detailed in the Methods section, the sampling and experimental procedures followed a specific schematic (Figure 1).

### 2.6. Statistics Analyses

Statistical analysis was conducted using Excel 2021 and SPSS 21.0 software for one-way ANOVA. The significance of the differences was assessed using Duncan’s new multiple range test and the least significant difference (LSD) method.

The bacterial count was determined by one-way ANOVA with flower source (2021–2023) and honey source (nectar, honey sac, and fresh honey) as factors and bacterial count and amylase values as dependent variables. Tukey’s HSD was used for the post hoc test.

## 3. Results

### 3.1. Sample Collection Results

The quantities of samples collected each year are as follows: for rape nectar, 50 mL was collected from a range of 4350 to 15,015 flowers; for honey sacs, 90 honey sacs were divided into nine centrifuge tubes; for honey sac fluid, 3 mL was obtained from 247 to 318 honey sacs of bees; and for honey, 15 mL was collected from 78 to 132 honeycomb cells. Feeding test samples: approximately 156–288 bees per colony received 3 mL of honey sac fluid for a total collection of 15 mL.

### 3.2. Abundant B. subtilis Were Detected in Nectar, Honey Sac, and Fresh Honey

**Figure 2:**
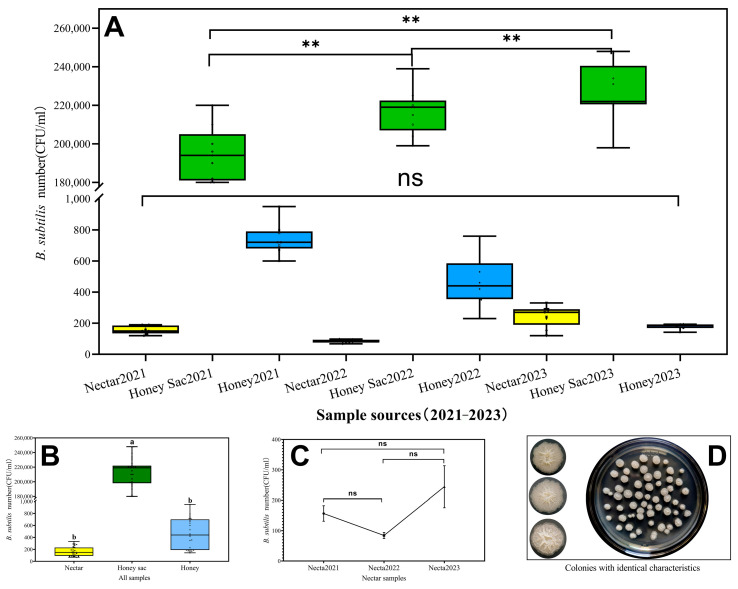
*B. subtilis* abundance in samples (mean ± SD) and colonies with identical characteristics. (**A**) Numbers of *B. subtilis* in the samples collected from nectar, honey sac, and honey during 2021–2023. (**B**) Numbers of *B. subtilis* in the combined samples across all three years. (**C**) Numbers of *B. subtilis* in nectar samples for each individual year. **—significant difference at *p* < 0.01. Boxes with different letters are significantly different (*p* < 0.05). ns—indicates no significant difference. (**D**) Colonies with identical characteristics.

Bacteria from samples collected over three years were counted, purified, and identified, and all bacterial colonies with identical characteristics were classified and randomly selected for 16S rRNA sequencing. *B. subtilis* colonies are grey-white and sometimes yellowish. They are round or irregular in shape, have a rough and opaque surface, and the colony size is usually about 2–5 mm, sometimes up to 3 cm. (Figure 2D). By BLAST comparison, *B. subtilis* was identified as the dominant species with abundance in all samples (Figure 2A and Table 1), while other bacteria were not listed due to their low abundance.

Statistical analysis revealed highly significant differences (*n* = 27,2021: F_2,24_ = 1786.717, *p* < 0.001; 2022: F_2,24_ = 3036.770, *p* < 0.001; 2023: F_2,24_ = 1954.405, *p* < 0.001) (Figure 2A). The *B. subtilis* counts in all samples were F_2,78_ = 3368.279 (*n* = 81), with *p* < 0.01 for rape nectar versus honey sac, indicating a highly significant difference, and *p* = 0.995 > 0.05 for nectar versus honey, indicating no significant difference (Figure 2B). The levels of bacteria varied significantly across nectar samples collected from different colonies over the years 2021 to 2023 (Figure 2C).

### 3.3. Molecular Bio-Identification of the Microbial Strains

During 2021–2023, three representative bacterial strains were obtained and designated AMHS2039, AMHS2785, and AMHS3076. BLAST gene sequence comparisons in GenBank revealed high similarity percentages and intervals, all greater than 99%. The sequences were submitted to NCBI and assigned accession numbers PQ288516, PQ288517, and PQ288518. Based on the average number of bacteria in the honey sac and the number of colonies grown on plates each year, the concentration and number of bacterial strains were simulated (Table 1).

Due to the large number of similar results identified from the bacterial strains, cluster analysis based on 16S rRNA was performed to determine the consistency of species and genus characteristics of the strains, and representative honey sac isolates were selected. Partial biochemical identification results (Table 2) were used, with negative (−) and positive (+) results, to finally confirm that all three bacterial strains were *B. subtilis*.

### 3.4. Higher Amylase Activities Were Detected in the Samples from Honey Sac and Honey Groups

**Figure 3:**
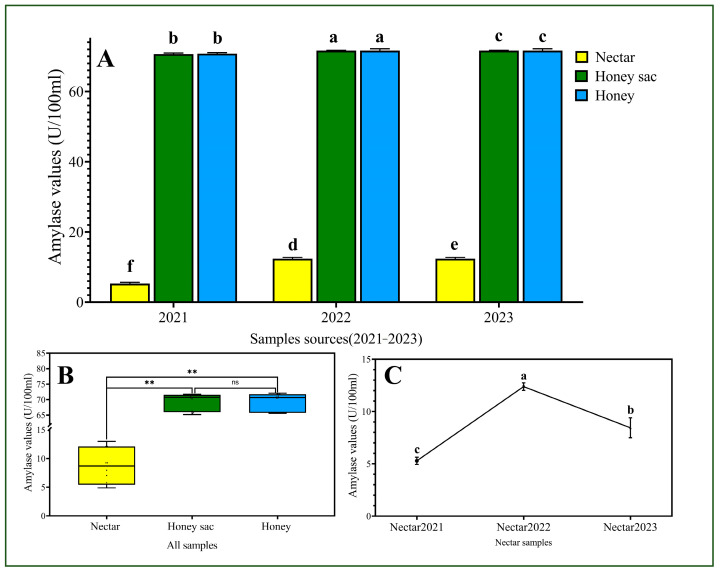
Amylase activities in the samples from nectar, honey sac fluid, and honey. (**A**) Amylase readouts from samples collected from nectar, honey sac fluid, and honey during 2021–2023. (**B**) Amylase readouts for the combined samples across all three years. (**C**) Amylase activity in nectar samples for each individual year. Bars and lines with different letters are significantly different (*p* ≤ 0.05). **—significant difference at *p* ≤ 0.01. ns—indicates no significant difference.

In 2021, the amylase values in the honey sac were 70.66 ± 0.27 U/100 mL, and in the honey, they were 70.76 ± 0.28 U/100 mL, with a *p* = 0.842 > 0.05, indicating no significant difference. In 2022, the AMS in honey sac was 71.62 ± 0.09 U/100 mL and in honey 71.62 ± 0.55 U/100 mL, with *p* = 1.000 > 0.05. Similarly, in 2023, the AMS in the honey sac was 65.70 ± 0.44 U/100 mL and in honey it was 65.70 ± 0.15 U/100 mL, with a *p* = 1.000 > 0.05. These results suggest no significant differences in AMS from honey sac to honey within each year. However, when comparing the differences in AMS between nectar and honey over the three years, a significant difference was observed. Our results showed that amylase levels varied among the different groups (Figure 3A). The amylase levels in the honey sac fluid and honey groups were significantly higher than those in the nectar group (Figure 3A,B). However, no statistical significance was observed between the honey sac fluid and honey samples over the three years. Amylase levels in the honey sac fluid and honey remained stable across the three years of collection, while the amylase levels in nectar showed significant variation. The amylase activity readouts from 2021 to 2023 fluctuated as follows: 5.28 ± 0.34 U/100 mL (2021), 12.38 ± 0.36 U/100 mL (2022), and 8.44 ± 0.94 U/100 mL (2023) (Figure 3C).

### 3.5. B. subtilis Can Produce Amylase in Culture When Provided with Sucrose or Nectar

In the absence of *B. subtilis* in culture, no amylase activity was detected in any control groups except the nectar group, which showed a moderate level of amylase activity. In the presence of *B. subtilis*, amylase activity was observed in all experimental groups. However, the group supplied with H_2_O and *B. subtilis* exhibited very low amylase activity and it is not significantly different from the amylase activities in the control groups. The highest amylase activity was found in the groups supplied with nectar and *B. subtilis* (Table 3). The groups supplied with various concentrations of sucrose and *B. subtilis* showed slightly lower amylase activity compared to the nectar-supplied groups, but significantly higher activity than the group supplied with water and *B. subtilis*. Among the sucrose groups, *B. subtilis* with 20% sucrose produced slightly higher amylase activity (Table 3).

Collectively, our data suggest that *B. subtilis* can produce amylase in culture when sucrose or nectar is present.

### 3.6. Measurement of the Amylase Activity Produced by B. subtilis in the Honey Sacs of Bees

To detect the amylase activity produced by *B. subtilis* in the honey sacs of bees, bees were fed with a variety of feeding materials. The bees fed with H_2_O comprise the control group. *B. subtilis* cannot produce amylase when supplied with H_2_O, which is supported by our simulation experiment. Therefore, the amylase activity from the honey sac in this group served as the baseline of the amylase activity in the bees. When supplied with sucrose or nectar, *B. subtilis* was able to produce amylase. The differences in amylase activity between the experimental groups (bees fed with sucrose or nectar) and the control group (bees fed with H_2_O) reflect amylase activity produced by *B. subtilis* in the honey sac. Compared to the control group, the amylase activity in the groups that were fed with either sucrose or nectar were higher, while the nectar group produced highest amylase activities (Table 4).

### 3.7. The Trend of Amylase Activity in Simulation and Feeding Experiments

**Figure 4:**
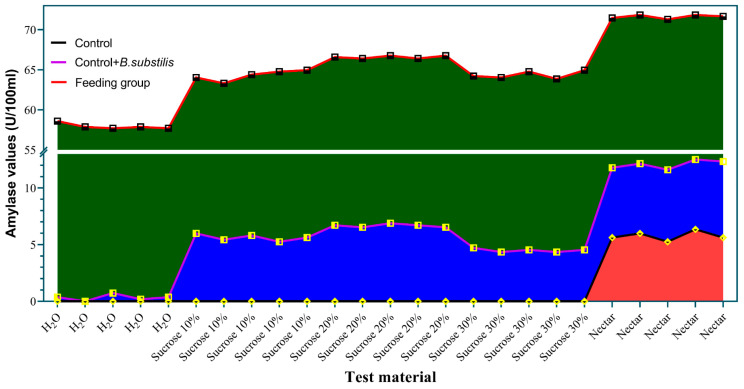
Trend line of amylase levels following simulated addition of *B. subtilis* and bee feeding. The black line represents the amylase levels measured from different supplement materials in the control group. The purple line indicates the change in amylase levels after *B. subtilis* was added to the supplement materials. The red lines represent the amylase values measured in honey sac fluid from bees that were fed with the different supplement materials. The red area highlights the amylase levels in the control group, while the blue area shows the difference in amylase levels between the *B. subtilis*-treated groups and the control group. The green area indicates the difference in amylase levels between the dietary and feeding groups.

To illustrate the trend of amylase activity in both the simulation and feeding experiments, the original data were plotted for comparison. The results show that the patterns of change across the three groups were essentially identical. Specifically, amylase levels following nectar feeding were significantly higher than those in the other groups, suggesting that the initial amylase levels influence amylase production in the honey sac fluid, with a statistically significant difference (Figure 4). Each experimental condition was repeated five times. The graph demonstrates that under each condition, the amylase activity readings were highly consistent, indicating that our testing system is stable and reproducible.

### 3.8. The Amylase Activity Produced by B. subtilis in the Honey Sac Aligns with the Amylase Activity Observed in the Simulation Experiment

To compare the amylase activity produced by *B. subtilis* in the honey sac with that produced by *B. subtilis* in culture, the amylase value in the honey sac was calculated using the following formula: For the samples from bees fed with sucrose: Amylase value produced by *B. subtilis* in the honey sac = Amylase value from the honey sac of bees fed with sucrose—Amylase value from the honey sac of bees fed with H_2_O. Since nectar itself contains a baseline level of amylase activity (Table 3), for the nectar group: Amylase value produced by *B. subtilis* in the honey sac = Amylase value from the honey sac of bees fed with sucrose—Amylase value from the honey sac of bees fed with H_2_O—Amylase value from nectar.

**Figure 5:**
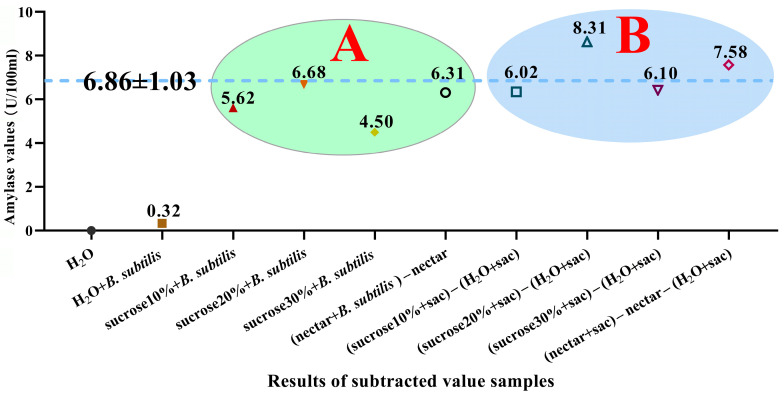
Trend plot of difference between simulation test and feeding results. The dashed blue line represents the difference between the mean amylase values of all samples (mean ± SD). (A) The green area is the difference in amylase values between the experimental group and its corresponding control group in the bacterial culture experiment. (B) The blue area is the amylase activity produced by B. subtilis in the honey sac of bees fed with each supplement material in the feeding experiment.

Our results indicate that the amylase value produced by *B. subtilis* in the honey sac is very similar to the value produced in culture (Figure 5). This similarity may be due to the fact that the number of *B. subtilis* used in the culture experiment was the same as the number present in the honey sac. Additionally, our in vitro culture system has proven to be stable and reproducible.

## 4. Discussion

This study was conducted over a three-year period from 2021 to 2023, with samples collected at the peak of rape flowering in late February each year. Due to inconsistent weather conditions each year, the timing of oilseed rape flowering varied slightly, as did the number of flowers per milliliter of nectar collected. However, the overall trend and significance of the bacterial counts and amylase levels in the samples collected over the three years were consistent. The results of this study indicate a high degree of correlation between amylase levels and bacterial populations over the years, suggesting a stable pattern despite the variability in collection conditions.

The low abundance of *B. subtilis* in nectar may be due to the fact that the buds of oilseed rape flowers are not exposed to the external environment prior to flowering and nectar secretion and are therefore unaffected by external factors such as temperature, humidity, environmental bacteria, and honeybee feeding [31]. In addition, the presence of hydrogen peroxide in the nectar can inhibit the growth of most bacteria [32]. The population of *B. subtilis* in nectar sacs remains relatively high throughout the collection year at a level of 10^5^ (CFU/g), indicating that the bacterial load in honeybees, nectar, and sacs is less influenced by external environmental factors, and once established, forms a relatively stable microbiota. The reduced bacterial load in honey can be attributed to the colonization of bacteria within the nectar sacs, coupled with the high sugar content of honey, which has a hypertonic effect that strongly suppresses bacterial growth.

Previous studies have shown that the carbohydrate composition of rape nectar is not affected by ploidy and other factors and is constant, consisting almost entirely of glucose and fructose [33]. These conditions allow for better control of the experimental conditions under the purer influencing factors. However, nectar is subject to fluctuations in amylase activity due to various factors such as climatic temperature, humidity, secretion levels, and bee collection [34]. Consequently, the amylase levels of honeydew samples collected over a three-year period from 2021 to 2023 showed highly significant differences (Figure 3C), with particularly high amylase levels observed in the 2022 samples. This increase was mainly attributed to the lower temperatures in that year, which were 2~4 °C cooler than in the other two years, resulting in longer bud life. In contrast, the amylase values in the samples from honey sac and honey were very stable across the three years. The amylase values in the honey sac and honey groups were much higher compared to the nectar group. This might be due to the influence of bee secretions, production by *B. subtilis*, and the inherent amylase values of the nectar itself.

Simulated experiments show that both *B. subtilis* and sucrose are required for amylase production. Amylase cannot be produced without either *B. subtilis* or sucrose in the culture system, indicating that both factors are necessary for amylase production. The previous literature has reported that *B. subtilis* produces amylase in response to various sugars, including glucose, fructose, lactose, and sucrose [35]. We also observed that a given amount of *B. subtilis* produces varying levels of amylase depending on the concentration of sucrose, suggesting that sucrose concentration may influence *B. subtilis*-dependent amylase production. Typically, bees are artificially fed sucrose in winter when there is a lack of outdoor nectar sources. Sucrose is a type of disaccharide formed by the condensation and dehydration of a molecule of glucose and a molecule of fructose, and it shares similarities with honey. Sucrose is also mentioned in the literature as the best substitute for nectar [36].

Feeding trials revealed that amylase levels in the honey sacs of bees fed with H_2_O were significantly lower than those in bees fed with sucrose solutions at varying concentrations, indicating that sucrose induces amylase production in *B. subtilis*. The highest amylase value was observed at a sucrose concentration of 20%, highlighting a clear influence of sucrose concentration on amylase induction. Furthermore, the amylase activity produced by *B. subtilis* in the honey sac closely resembles the amylase activity produced in our in vitro bacterial culture system, suggesting that our simulation experiment accurately mimics the in vivo conditions of *B. subtilis* amylase production in the honey sac.

As mentioned above, it was previously thought that all honey amylase was secreted by bees, but it was only considered that bees as living organisms secreted amylase and eventually mixed into honey, which was a limitation of previous studies. As we study bee gut microbes, we learn more about the types of microbes that affect bee health and products. Therefore, this study also fills the research gap in one aspect. Tomas Erban’s research uses a single proteomic dataset to identify foreign amylases in adulterated honey and proposes the use of different protein markers to detect such adulteration [37]. However, in adulterated honey, bacterially produced amylases are typically used for adulteration. There is a potential risk of misinterpretation when relying solely on proteomic identification of adulterated amylases in honey, especially when considering the amylases produced by *Bacillus amyloliquefaciens* and *Bacillus licheniformis* mentioned in the article, due to the possibility of the presence of naturally occurring bacterial amylases in honey. Therefore, this study also provides a basis for future research directions.

Building on the findings of this study, future research efforts could be directed towards honey proteomics, with a focus on investigating the origins and differences in levels of amylase in honey sacs and honey in relation to bacterial amylases. This approach will serve as the basis for further studies to provide a comprehensive analysis of the proteomic landscape to elucidate specific contributions and variations in the enzyme profile in these bee products.

## 5. Conclusions

This comprehensive study provides valuable insights into the dynamics of amylase production in bees, challenging the long-held belief that amylase is solely a product of bee secretions. Our findings demonstrate a significant presence of *B. subtilis* in nectar, the honey sac, and honey, with particularly high concentrations in the honey sac. This bacterial presence, quantified at up to 10^5^ CFU/g, indicated that *B. subtilis* had a potential effect on bee sac enzyme activity.

Our results indicate that the amylase activity in honey is not only a reflection of bee activity but also influenced by microbial activity within the hive, particularly by *B. subtilis*. This discovery has important implications for the standardization and quality assessment of honey, suggesting that future regulations and freshness indicators should account for the role of microflora in addition to traditional measures.

In conclusion, this study expands our understanding of the enzymatic profile of honey, introducing a new perspective that highlights the symbiotic relationship between bees and their associated microorganisms.

## Figures and Tables

**Table 1:** Molecular bio-identification of three strains of *B. subtilis* and concentrations of *B. subtilis* used for simulation experiment.

Year	Isolation and Accession Number of NCBI	Similar Bacterial Strain and Login Number of NCBI	Similar Interval	Sequence Lengths and Similarity	Concentration of *B. subtilis* Used in Simulation Experiment (Total Number 1000 µL)
2021	AMHS2039 (PQ288516)	*B. subtilis* strain SEM2H8 16S ribosomal RNA gene, partial sequence (MW380566.1)	19–1355	1329/1338 (99%)	10^−5^, 428 μL
2022	AMHS2785 (PQ288517)	*B. subtilis* strain 4I3 16S ribosomal RNA gene, partial sequence (MW380592.1)	8–1449	1442/1442 (100%)	10^−5^, 479 μL
2023	AMHS3076 (PQ288518)	*B. subtilis* strain FQ32 16S ribosomal RNA gene, partial sequence (MF144479.1)	37–1336	1300/1300 (100%)	10^−5^, 498 μL

**Table 2:** Results of biochemical identification of three strains of *B. subtilis*.

0~11	Culture Time	0	GLY	ERY	DARA	LARA	RIB	DXYL	LXYL	ADO	MDX	GAL	GLU
AMHS2039	24 h	−	+	−	−	+	+	+	−	−	−	−	+
	48 h	−	−	−	−	+	+	+	−	−	−	−	−
AMHS2785	24 h	−	+	−	−	+	+	+	−	−	−	−	+
	48 h	−	−	−	−	−	−	−	−	−	−	+	−
AMHS3076	24 h	−	+	−	−	+	+	−	−	−	−	−	+
	48 h	−	−	−	−	−	−	+	+	+	+	−	−

The first line is the results from 24 h culture, and the second line is the results from 48 h culture. The first line is the main result, and the second line is the reference. GLY (mannitol), ERY (erythritol), DARA (D-arabinose), LARA (L-arabinose), RIB (d-ribose), DXYL (D-xylose), LXYL (L-xylose), ADO (D-lateral calendulin), MDX (methyl-D-pyranoside), GAL (D-galactose), GLU (D-glucose).

**Table 3:** Amylase activities in the groups of the simulation experiment.

Groups	Test Material	Amylase Values (U/100 mL) (Mean ± SD) Significance
Control groups	H_2_O	0 (a)
	10% Sucrose	0 (a)
	20% Sucrose	0 (a)
	30% Sucrose	0 (a)
	Nectar (Control)	5.77 ± 0.41 (b)
Experimental groups	H_2_O + *B. subtilis*	0.32 ± 0.26 (a)
	Sucrose 10% + *B. subtilis*	5.62 ± 0.28 (b)
	Sucrose 20% + *B. subtilis*	6.68 ± 0.15 (c)
	Sucrose 30% + *B. subtilis*	4.49 ± 0.15 (d)
	Nectar + *B. subtilis*	12.08 ± 0.38 (e)

The letters in brackets, listed in the column showing amylase values, represent statistical significance among the groups. Groups with the same letter indicate no significant difference (*p* > 0.05), while groups with different letters indicate a significant difference (*p* ≤ 0.05).

**Table 4:** Group amylase values from the feeding trials.

Groups	Feeding Materials	Amylase Values (U/100 mL) (Mean ± SD) Significance
Control group	H_2_O	57.94 ± 0.37 (a)
Experimental groups	10% sucrose	64.29 ± 0.64 (b)
	20% sucrose	66.57 ± 0.18 (c)
	30% sucrose	64.36 ± 0.46 (b)
	Nectar	71.61 ± 0.23 (d)

The letters in brackets, listed in the column showing amylase values, represent statistical significance among the groups. Groups with the same letter indicate no significant difference (*p* > 0.05), while groups with different letters indicate a significant difference (*p* ≤ 0.05).

## Data Availability

Data available on request from the corresponding authors. The data underlying this article are available in the GenBank Nucleotide Database at NCBI and can be accessed by using the accession number.
